# Collective screening tools for early identification of dyslexia

**DOI:** 10.3389/fpsyg.2014.01581

**Published:** 2015-01-23

**Authors:** Olga V. C. A. Andrade, Paulo E. Andrade, Simone A. Capellini

**Affiliations:** ^1^Department of Pedagogical Studies, School of Elementary and Secondary Education “Colégio Criativo,” MaríliaBrazil; ^2^Department of Speech and Hearing Sciences, São Paulo State University “Júlio de Mesquita Filho” – Faculdade de Filosofia e Ciências/Universidade Estadual PaulistaMarília, Brazil

**Keywords:** language, phonological processing, reading, writing, children, early literacy

## Abstract

Current response to intervention models (RTIs) favor a three-tier system. In general, Tier 1 consists of evidence-based, effective reading instruction in the classroom and universal screening of all students at the beginning of the grade level to identify children for early intervention. Non-responders to Tier 1 receive small-group tutoring in Tier 2. Non-responders to Tier 2 are given still more intensive, individual intervention in Tier 3. Limited time, personnel and financial resources derail RTI’s implementation in Brazilian schools because this approach involves procedures that require extra time and extra personnel in all three tiers, including screening tools which normally consist of tasks administered individually. We explored the accuracy of collectively and easily administered screening tools for the early identification of second graders at risk for dyslexia in a two-stage screening model. A first-stage universal screening based on collectively administered curriculum-based measurements was used in 45 7 years old early Portuguese readers from 4 second-grade classrooms at the beginning of the school year and identified an at-risk group of 13 academic low-achievers. Collectively administered tasks based on phonological judgments by matching figures and figures to spoken words [alternative tools for educators (ATE)] and a comprehensive cognitive-linguistic battery of collective and individual assessments were both administered to all children and constituted the second-stage screening. Low-achievement on ATE tasks and on collectively administered writing tasks (scores at the 25th percentile) showed good sensitivity (true positives) and specificity (true negatives) to poor literacy status defined as scores ≤1 SD below the mean on literacy abilities at the end of fifth grade. These results provide implications for the use of a collectively administered screening tool for the early identification of children at risk for dyslexia in a classroom setting.

## INTRODUCTION

Dyslexia is a neurologically based learning disorder with a genetic origin (Vellutino et al., 2004; Shaywitz et al., 2008; Peterson and Pennington, 2012) defined as “difficulties in accuracy or fluency of reading that are not consistent with the person’s chronological age, educational opportunities, or intellectual abilities” (Snowling and Hulme, 2012, p. 594); prevalence in the general population varies from 5 to 17% according to language, orthography transparency and with the criteria used to define low-achievement on reading ability (Ziegler and Goswami, 2005; Shaywitz et al., 2008). There is a great body of evidence since Bradley and Bryant’s (1983) study that phonological processing abilities, mainly phonological awareness (PA), rapid naming (RN), and verbal working memory (VWM), are the strongest early predictors of literacy acquisition (Fletcher et al., 2002; Fuchs et al., 2012) whose difficulties are considered by many as the primary cause of dyslexia (Ramus, 2003; Vellutino et al., 2004; Snowling and Hulme, 2012). However, there is also cogent evidence that RN can be an independent risk factor with separate and unique predictive power (Wolf and Bowers, 1999; Boada et al., 2012). Additionally, children with attention deficit hyperactivity disorder (ADHD) frequently show reading problems and a growing body of research has shown that inattention symptoms are associated with weaknesses in naming speed and working memory (Shanahan et al., 2006; Lui and Tannock, 2007; Marzocchi et al., 2008; Arnett et al., 2012), especially working memory tasks requiring manipulation of information with a higher demand on the central executive mechanisms (Kasper et al., 2012) and mainly those involving visuospatial manipulations (Martinussen et al., 2005; Willcutt et al., 2005; Castellanos et al., 2006). In sum, there is an emergent consensus that inattention *per se* is associated with RN and working memory deficits and, hence, is a risk factor for dyslexia regardless of the presence or absence of PA impairment (Shanahan et al., 2006; Shaywitz and Shaywitz, 2008; Arnett et al., 2012).

In an attempt to capture the unexpected nature of reading difficulties emphasized in the definitions of dyslexia, the traditional clinical approaches have sought to operationalize its identification/diagnostic in terms of an IQ-achievement discrepancy, which requires reading scores of one or one-and-one-half standard deviation below standard scores on IQ for a child to be considered learning-disabled (Fuchs and Fuchs, 2006). However, the IQ-discrepancy model has been frequently criticized for two main reasons. Firstly, even if a student show clear difficulties since the early days of learning to read, he/she must wait until the third year or more (typically until the fifth grade) so that student’s achievement is sufficiently low and the discrepancy between IQ and achievement becomes large enough to allow the diagnostic and, hence, the student can be considered eligible for intervention. Secondly, research has failed to find qualitative differences in reading performance and cognitive abilities between low-achievers who fulfill the requirement of discrepancy and low-achievers who do not, but according to discrepancy model only the first ones will receive the intervention they need. Therefore, the discrepancy model is deemed unfair because it deprives of necessary assistance those children who are as needy and deserving as those given the label (Fuchs and Fuchs, 2006, p. 96). For these and other reasons, in the reauthorization of the Individuals With Disabilities Education Improvement Act allowed practitioners to use a new, alternative, model of identification called “response to intervention” (RTI), an evidence-based multi-tiered system of preventative intervention and progress monitoring in which all children are first provided with research-based instruction and universal screening of students at risk for dyslexia, whose progress is frequently monitored (Fuchs and Fuchs, 2006, 2007; Fletcher and Vaughn, 2009).

Researchers have currently favored RTI models of three tiers (Fuchs and Fuchs, 2006, 2007; Davis et al., 2007; Fletcher and Vaughn, 2009). In general, Tier 1 consists of evidence-based, effective reading instruction in the general education classroom and universal screening of all students at the beginning of the school year to identify children for early intervention. Those identified as at-risk have their progress monitored for a short time (e.g., 5 weeks) to confirm/disconfirm the risk status. Non-responders to Tier 1 are referred to Tier 2 which consists in small-group tutoring of three to five students for 20–40 min daily, 3 to 4 times per week over 15- to 20-week sessions (Fuchs and Fuchs, 2007; Fletcher and Vaughn, 2009). Non-responders to Tier 2 are deemed unexpected low-achievers and then are referred to Tier 3, the most intensive form of instruction and already considered special education (Fuchs and Fuchs, 2007; Fletcher and Vaughn, 2009), where they are given still more intensive, individual, and increased intervention (45–60 min or up to 1.5 h per day) with a more specialized teacher (Fuchs and Fuchs, 2007; Fletcher and Vaughn, 2009).

It is generally agreed that universal screening for at-risk students is a key component of RTI models which plays a crucial role, both in terms of prevention and identification, for a successful implementation of RTI (Davis et al., 2007; Fletcher and Vaughn, 2009; Fuchs et al., 2012; Gilbert et al., 2012). An accurate classification should yield a high percentage of true positives (TPs; children correctly identified as at risk, i.e., who will indeed show persistent difficulties in learning to read) and a high percentage of true negatives (TNs children correctly identified as not at risk, i.e., who will not have reading difficulties). Although the ideal would be to have a classification accuracy of 100% of sensitivity and specificity, no screening system (one or more screening tools used to identify at risk children) reaches these ideal values so that “false positives (FPs) and false negatives (FNs) are inherent to any assessment device and are inevitably linked” (Fletcher et al., 2002, p. 55). FPs result from incorrect identification of children who score below the cut score on the screening instrument, but are not actually at risk and soon become good readers. FNs, on the other hand, occur when some children perform well on the screening test and are not identified as at risk, but who are actually at risk and soon will become struggling readers. Sensitivity is the term used to designate the proportion of TPs whereas specificity refers to the proportion of TNs a given screening system is capable to identify. Sensitivity is calculated by dividing the number of TPs by the sum of TPs and FNs [Sensitivity = TP/(TP+FN)] whereas specificity is obtained by dividing the number of TNs by the sum of TNs and FPs [Specificity = TN/(TN+FP); Vellutino et al., 2008; Fuchs et al., 2012].

Since FPs and FNs are inherent in all screening measures (Fletcher et al., 2002), then accuracy is necessarily a result of the trade-off between sensitivity and specificity (Fuchs et al., 2012; Gilbert et al., 2012). Therefore, accuracy is the proportion of TPs and TNs to the whole sample (*N*) calculated by dividing the sum of TPs and TNs by the whole sample [Accuracy = (TP+TN)/*N*]. An important rationale underlying this trade-off is that FNs mean the number of children actually at risk who will not receive the intervention they need, whereas FPs mean the number of children not at risk who will receive extra instruction (Fletcher et al., 2002; Gilbert et al., 2012). Although FPs are of concern because they imply increased costs to already costly interventions, the FNs are much less desirable (Fletcher et al., 2002; Barth et al., 2008).

Since universal screening involves all students in a school it is imperative “that the tool can be quickly administered with adequate sensitivity and specificity” (Fletcher and Vaughn, 2009, p. 32). Traditionally, RTI approaches implement screening using curriculum-based measurements (CBMs), typically a single word reading measure administered individually at the beginning of the school year (Fuchs and Fuchs, 2006; Fletcher and Vaughn, 2009). The selection of a critical cutoff score to define at-risk performance vary from more conservative cut-points, such as 1 to 2 standard deviation (SD) below the mean, to more liberal, such as 25th and 30th percentiles, which correspond approximately 0.68 SD and 0.5 SD, respectively (Catts et al., 2001; Fletcher et al., 2002; Fuchs and Fuchs, 2006; Davis et al., 2007; Fletcher and Vaughn, 2009). Stricter cut-points decrease the probability of FPs while increase the probability of FNs, whereas more liberal cut-points tend to produce the opposite, i.e., to decrease FNs (producing more TPs) while increase FPs (Fletcher et al., 2002; Davis et al., 2007; Barth et al., 2008). Although FNs are a less desirable error than FPs because it excludes children in need of intervention, an inflated proportion of TPs implies in delivering costly prevention to students who do not need it thus stressing the already scarce school resources (Davis et al., 2007; Barth et al., 2008; Fuchs et al., 2012). While it has been suggested that an acceptable sensitivity and specificity should be around 90 and 85%, respectively (Vellutino et al., 2008), research has shown that 1-time universal screening has produced an unacceptably high proportion of FPs, ranging from 20 to 60% (Vellutino et al., 2008), whereas specificity can range from 20 to ∼60% when sensitivity is held constant (Johnson et al., 2009, 2010).

In order to maximize the number of TPs while limiting the number of FPs it has been proposed that screening for at-risk students should be made in a two-stage process (Catts et al., 2001; Compton et al., 2010; Fuchs et al., 2012). The first-stage consists of a universal screening based on a brief individual assessment (normally a measure of reading fluency) using a more lenient cut point (e.g., 30th percentile) in order to maximize TPs, thus excluding most of the TNs from the costly Tier 2 intervention. Children identified as at-risk in the first stage have their progress monitored during a period of about five or 6 weeks of primary intervention, after which a second second-stage screening consisting of a more thorough assessment is conducted, so that only those identified as at-risk in the second-stage screening are referred to secondary intervention (Compton et al., 2010; Fuchs et al., 2012). For instance, Compton et al. (2010) used the Word Identification and Word Attack subtests of the Woodcock Reading Mastery Tests and the Sight Word Efficiency and Phonemic Decoding Efficiency subtests of the Test of Word Reading Efficiency in the first-stage, universal screening of at-risk students in a sample of 355 children in the fall of first grade. They found that the use of phonemic decoding efficiency (the number of nonsense words accurately decoded in 45 s) as the first-stage screening tool and 6 weeks of progress monitoring with word identification fluency (WIF) and dynamic assessment as the second-stage screening eliminated 43.4% of FPs of the sample, being the most efficient of the four two-stage models tested.

More recently, Fuchs et al. (2012) have tested five two-stage screening models in 783 children from 42 first-grade classrooms in 16 schools. In the first stage, the six lowest scores on WIF and rapid letter naming of each class (∼30th percentile) were identified as at-risk, totaling 252 students (32% of the sample), of whom only 195 remained until the end of the study. Then, the authors tested two types of screening tool for their second-stage screening. One type was a cognitive battery administered in the early fall consisting of multiple tests measuring de cognitive dimensions of RN, phonological processing, oral language comprehension, and non-verbal reasoning. The second type of a second-stage screening consisted of 18 weeks of WIF assessments from which authors modeled December and May reading outcomes. From the combination of these two types of second-stage screening the researchers derived five models of second stage-screening. The two models based solely on December and May reading performance resulted in the two lowest specificities (26.5 and 43.9%, respectively). Logistic regression analyses showed that the two models derived from the combination of the cognitive measures with WIF May and December intercepts, respectively, were the two most accurate models to predict fifth-grade reading comprehension when sensitivity was held constant at 91.7%, with specificities of 68.4% for the May intercept and of 67.3% for the December intercept. The most interesting finding, however, was that the model based solely on the cognitive measures showed a comparable fit, with a specificity of 67.3%. This model is less expensive and can be implemented already in the first 2 months of the school year, being more parsimonious and, hence superior. The use of a second-stage screening based only on the cognitive measures reduced the number of students in need of tutoring from 195 identified in the first stage to 65, thus eliminating 66.7% of FPs. From the 65 students identified as at risk in the second stage 32 were FPs and 33 were identified as dyslexics in fifth grade, what represented significant savings for school districts.

Educational resources in Brazil, however, are still much scarcer than in developed countries, either in time, personnel, and financial resources. This set of unfavorable factors seriously derails RTI’s implementation in Brazilian schools and makes developing strategies to overcome these limitations an obligatory aim.

The present investigation is a pilot study in which we explored the utility of two-stage screening models based solely on collective screening tools in a non-clinical school sample of 45 Brazilian second graders. Curriculum-based assessments (CBAs) carried out in the classroom for all students 2 weeks after the start of the school year were used as the universal screening of the first stage. As part of the second stage we administered a cognitive-linguistic protocol (Capellini et al., 2012) and a set of collective screening tools based on phonological judgments by matching figures and figures to spoken words as well, hereinafter referred to as alternative tools for educators (ATEs). The Capellini et al. (2012; CS&S) protocol was developed in Brazil with the purpose to identify the cognitive-linguistic profile of Brazilian children in the first stages of reading. ATE tasks were also developed in Brazil for the collective assessment of phonological abilities by the first author (Andrade et al., 2011).

Firstly, we performed a principal components analysis (PCA) factor using a varimax rotation including all measures of the C&S protocol and ATE tasks to examine the factor structure of the CS&S protocol and check whether results actually suggest its proposed cognitive-related dimensions (e.g., literacy, phonological processing, VWM, and visual and visuospatial working memory). PCA also provided the extent and nature of relationships between ATE tasks and the cognitive and linguistic measures. Composite scores based on summed raw scores of writing, reading, phonological and naming abilities were created and used to obtain the percentiles and cutoff points to identify low-achievers, as well as to compare performances of low-achiever and non low-achiever on cognitive-linguistic measures and ATE tasks. Although tasks with larger standard deviation will be weighted more when summing raw scores, we used this procedure in place of summed z-scores for its practical value for educators.

Finally, composites scores were also used to run regressions analysis in order to explore the predictive power of the collective tools (ATE tasks and C&S writing composite scores) at the end of fifth grade and to run logistic regression analysis in order to obtain the sensitivity, specificity, and overall accuracy of different combinations of these collective screening tools in the early identification of poor literacy status at the end of fifth grade level.

## MATERIALS AND METHODS

### PARTICIPANTS

This prospective, longitudinal study was carried out with a non-referred school-based sample of 69 early Portuguese readers, corresponding to all students enrolled in four second-grade classrooms of elementary education (as per the grade distinctions in the Brazilian education system) in an upper-middle class private school. However, considering children who could not participate because exclusionary factors (sensory deficits, neurological abnormality, known syndrome, etc.) and those who were granted permission by their parents to participate in this study, we were left with 45 students, of which 41 were right-handed. Participants were native speakers of Brazilian Portuguese and had no major sensorimotor handicaps (deafness, blindness) or pervasive neurodevelopmental disorders (psychosis, autism) or inadequate command of their native language. Age was calculated at the onset of test administration ranging from 6 to 8 years (average: 7 years and 2 months, SD: 4 months).

Although this sample size is small when it comes to a study on screening students at risk for a disorder, it is still a reasonable one for a pilot study if we take into account that we wanted a sample with certain specific characteristics, including homogeneity with respect to age, socioeconomic status, and pedagogical approaches. Additionally, we argue that a good, effective screening system should show consistent results with either relatively small samples or large samples. In fact, RTI approaches usually adopt a normative cut point, referenced to the classroom, school, district, or nation, below which students are designated at-risk (see Fuchs and Fuchs, 2006; Fuchs et al., 2012). Typically, RTI systems employ universal screening whereby all children in a school are assessed at the beginning of the school year with a critical norm-referenced cut-point on certain measures (usually ≤25th percentile). Furthermore, one important recent study (Fuchs et al., 2012) have used a cut point referenced to the classroom by selecting the six lower scores of each class from 42 first-grade classrooms in 16 schools what corresponds approximately to 25th to 30th percentile, for the first stage of a two-stage screening process.

### BEHAVIORAL MEASURES

Participants were first evaluated in their academic achievement using CBAs, in their cognitive-linguistic abilities using Cognitive-Linguistic Protocol by Capellini et al. (2012) and in their phonological abilities by using the collective phonological tasks referred to as ATEs (Andrade, 2010; Andrade et al., 2011, 2013). Participants were also screened for ADHD symptoms.

#### Curriculum-based assessments

Academic achievements were used as a first-stage universal screening and evaluated during the four first weeks after the start of the school year by the school’s pedagogical team. The school to which belonged the participants of the present study adopts a procedure which in Brazil is called “diagnostic assessment” (Luckesi, 2005), i.e., a comprehensive set of curriculum-based tests applied at the beginning of all grades and at the outset of a unit of study, aimed at evaluating the student’s learning achievements and needs. According to Luckesi (2005) assessments in the school should change from being purely summative to be eminently “diagnostic,” i.e., assessments systematically built in to the curriculum with the aim of identifying the strengths and weaknesses of the students in order to make decisions about further teaching strategies and/or specific interventions, if necessary.

Therefore, the diagnostic assessment adopted in the present paper is somewhat similar to the CBM methods (Fuchs and Fuchs, 2006; Fletcher and Vaughn, 2009), also referred to as CBAs (Dombrowski et al., 2004), used in the RTI approaches “to tailor instructional–intervention decisions to more appropriately suit the learning needs of the student” (Dombrowski et al., 2004, p. 367). Accordingly, all participants received curricular pedagogical activities specially designed for the present screening procedure and collectively administered to assess performance on core academic achievement areas. For purposes of this study, however, our main focus will be on assessments of reading and writing skills (Fuchs and Fuchs, 2006; Fletcher and Vaughn, 2009).

Assessments of literacy abilities in this diagnostic assessment included:

(i) Reading
–fluency and accuracy in reading aloud a list of simple monosyllabic, disyllabic, and trisyllable words, extracted from the textbooks used by the school
–draw the meaning of a written word (silent reading)
(ii) Writing
–letter knowledge: write all the known letters of the alphabet (print or cursive letters); make a circle with red pencil around the vowels and blue pencil around the consonants; write the letter with which begins the name of a picture
–syllable knowledge: write the correct syllables to complete the word
–word knowledge: using the mobile alphabet form as many words as you know and then write them on the paper
–spelling: write the dictated words
–oral language comprehension: draw what you understood of the story you just listened to.

#### Psychometric measures

The Cognitive-Linguistic Protocol (CS&S) was developed in Brazil with the aim of producing an effective instrument to identify the cognitive-linguistic profile in the first stages of reading acquisition of Brazilian children. The CS&S was administered 6 weeks after the start of the school year, and the CS&S measures we used in the present study and their respective scoring criteria are outlined below:

#### Alphabet task (collective administered)

Students were asked to recall all the letters of the alphabet in written form. There was no prompting or assistance. Scores were out of 26: the number of correct letters the child wrote down. The task was untimed.

#### Reading tasks (individually administered)

(i) *Reading fluency:* Number of words read aloud and audibly by the student in 1 min from a list of 70 words. If reading of all 70 words last less than 1 min the number of words correctly read was estimated for 1 min. Thus, the maximum score depended ultimately on a child’s reading speed.
(ii) *Reading accuracy:* In the same trial of reading fluency the number of words read aloud correctly from the same list of 70 words (untimed), with a maximum score of 70.
(iii) *Reading non-words*: Number of non-words read aloud correctly from a list of 10 (untimed). Maximum score of 10.

#### Writing tasks (collectively administered)

(i) *Writing words:* Number of words written with correct spelling from a list of 30 presented aurally (untimed and collectively administered in the classroom). Maximum score of 30.
(ii) *Writing pseudowords:* Number of pseudowords written with correct spelling from a list of 10 presented aurally. Maximum score of 10. Task was untimed.

#### Phonological tasks (individually administered)

(i) *Alliteration:* Matching first sounds in spoken words. Children are presented with three words spoken by the examiner and then are asked which of three spoken words start with the same sound. After three practice items, the test comprises 10 items leading to a maximum score of 10.
(ii) *Rhyme detection:* Matching last sounds in spoken words. Children are presented with three words spoken by the examiner and then are asked which of three spoken words end with the same sound. After three practice items, the test comprises 20 items leading to a maximum score of 20.
(iii) *Syllable segmentation:* To repeat the word spoken by the examiner while tapping to each syllable. Correct syllable breakdown of 12 words leads to a maximum possible score of 12.
(iv) *Auditory word discrimination:* To say whether a pair of words, spoken by the examiner and eventually differing in a single phoneme, are the same or different. Maximum possible of score of 19 for 19 trials.

#### Verbal working memory (individually administered)

(i) *Word sequence:* Number of correctly repeated two to five-word sequences (two unique trials for each sequence with the exception of the first example) spoken by the examiner in an interstimulus interval of 1 s. Familiar two or three-syllable words were presented. Score was given by the number of sequences correctly accomplished, with a maximum score of 7.
(ii) *Non-word repetition:* Number of correctly repeated non-words from a list of 23 presented aurally by the examiner. Maximum score of 23 for non-words spoken correctly.
(iii) *Verbal number sequence backward:* Number of correctly repeated two to five-number sequences backward (two trials for each sequence) that were spoken by the examiner. Maximum score of 8.

#### Shapes copying (collectively administered)

Correctly copied four archetypal forms: a circle, a square, a diamond, and a complex abstract figure. Students were able to see shapes while copying was performed. Students were only allowed to erase work on the last shape. Figures were scored through comparison with a standardized table of different visual representations of the shapes and measured with a diagramed seven-point scale. Maximum score for the task was 7.

#### Visual short term memory (individually administered)

This task was based on the Benton Visual Retention Test (BVRT; Sivan, 1992). In the BVRT’s original version, one or more simple geometric figures are drawn from memory after a brief exposure (typically 5 s for adults and 10 s for children), and two types of scores are computed: the number of correct representations and the number of errors. The visual short-term memory task from CS&S protocol is different from BVRT in that, instead of drawing from memory, children receive the same figures they just have seen, on cards in scrambled order, and have to organize them in the same order and position.

(i) *Figure order*: Ordering of solid abstract figures, i.e., after seeing two to five-figure sequences over a 10 s period, student was asked to reassemble the ordered pattern of figures in the same order and rotation. Score is given by the number of sequences in which the figures were correctly ordered, with a maximum score of 8.
(ii) *Figure rotation error:* Number of errors made in rotation of the shapes in figure order task. 28 figures were presented throughout the sequences, leading to a maximum of 28 errors.
(iii) *Figure rotation hit:* Number of hits made in rotation of the shapes in figure order task. 28 figures were presented throughout the sequences, leading to a maximum of 28 hits.
(iv) *Note:* Rotation hit is a way to measure the number of correct rotations proposed by Andrade et al. (in preparation) as a “positive measure” which provides information about the visuospatial working memory capacity of all subjects. While rotation error score allows only the identification of those who likely have impairments in visuospatial working memory, rotation hit provides information about each individual’s performance on this ability and in relation to the whole sample.

#### Rapid naming (individually administered)

(i) *Rapid naming (Figure)* of a list of four objects (house, ball, elephant, clock) displayed pictorially in a particular order. A different ordering was presented in each trial, with 10 total trials. Total time was recorded in seconds.
(ii) *Rapid naming (Number)* of numbers one through nine as listed visually in a random order. Screening performed before task to confirm that the child knew numbers one through nine. Time was recorded in seconds.

#### Alternative tools for educators

Consisted of phonological judgments by matching figures and figures to spoken words, developed by Andrade (2010) and Andrade et al. (2011) specifically with the goal of providing an effective non-literacy based tool for the collective assessment of the phonological abilities.

#### ATE I-alliteration

Children are presented with three words, depicted as three pictures, and are asked to mark with “X” the two pictures whose name start with the same sound. After three practice items, the test comprises 10 items leading to a maximum score of 10.

#### ATE II-alliteration

After matching a word presented aurally by the examiner with three words depicted as pictures, children are asked to mark with “X” the picture whose name starts with the same sound as the spoken word. After three practice items, the test comprises 10 items leading to a maximum score of 10.

#### ATE III-rhyme

Children are presented with three words, depicted as three pictures, and are asked to mark with “X” the two pictures whose name end with the same sound. After three practice items, the test comprises 10 items leading to a maximum score of 10.

#### ATE IV-rhyme

After matching a word presented aurally by the examiner with three words depicted as pictures, children are asked to mark with “X” the picture whose name ends with the same sound as the spoken word. After three practice items, the test comprises 10 items leading to a maximum score of 10.

#### ADHD symptoms

Classification of children as at-risk for ADHD (AR-ADHD) was based on pedagogical team (including teachers and the pedagogical coordinator) endorsements on the Diagnostic and Statistical Manual of Mental Disorders, Fourth Edition (DSM-IV; American Psychiatric Association, 2000) ADHD symptom checklist. DSM-IV checklist is an 18-item scale in which inattentive and impulsive behaviors are rated on a 4-point Likert scale (0 = not at all, 1 = just a little, 2 = pretty much, and 3 = very much). Children with ≥6 “pretty much” or “very much” ratings in either of the two domains (inattention or hyperactivity/impulsivity) were classified as “at-risk” for ADHD (AR-ADHD). Endorsements on ADHD checklist by the pedagogical team were further confirmed by interviews with parents.

### PROCEDURE

The initial sample of 69 children was screened for reading and writing difficulties using CBAs 2 weeks after the start of the school year as the first stage, universal screening, of a two-stage screening process. One month later, we administered the CS&S protocol and the ATE tasks and screened the entire group for ADHD symptom as well, as a part of the second stage. ATE tasks and the collective subtests of the C&S protocol were administered to all participants concurrently in the classroom, followed by individual administration of the linguistic and cognitive tests over the course of 6 weeks. Particularly, the accuracy with which CS&S writing tasks and ATE tasks predicted end of fifth grade level poor literacy status was investigated.

All study participation took place during school hours, so classroom administration was implemented for time efficiency on tests that did not require one-on-one monitoring. Testing began at the beginning of the academic calendar year.

After excluding children because exclusionary factor and after returning the consent form (approved by the “Ethics Committee from the Faculty of Science and Philosophy of São Paulo State University “Júlio de Mesquita Filho” – Faculdade de Filosofia e Ciências/Universidade Estadual Paulista, Marília, São Paulo, Brazil, under the protocol 0630/2009) we were left with 45 participants. Data collection occurred over the course of 3 years at three separate time intervals (T), namely, at the beginning of the academic calendar year of the second (T1) and third (T2) grades and at the end of fifth grade (T3) grades. However, we will restrict our analysis only in the T1 and T3 periods. In addition to the administration of the CS&S protocol screening, educators’ endorsements on DSM-IV ADHD symptom checklist were also carried out at each of the three time intervals and by different teachers, once there were different teachers for each grade level.

### STATISTICAL ANALYSIS

A PCA factor using a varimax rotation was performed on the measures of C&S protocol and ATE tasks as well (**Table 1**). Although our sample size is considered small when it comes to PCA, thus requiring caution in interpretation, the generation of high dimensional data with small sample sizes [i.e., High Dimension, Low Sample Size] is fast becoming a common place occurrence in many areas of modern science, and a growing body of literature has shown that some rules of thumb in the factor analytic literature, such as recommendations on absolute N (ranging from 50 to 200) and the number of cases per variable or N/p ratio (ranging from 3 to 20) are inconsistent. Actually, when communalities are high and the number of factors is small, factor analysis can be reliable for sample sizes well below 50 (Mundfrom et al., 2005; Henson and Roberts, 2006; de Winter et al., 2009). Anyway, we reiterate that this is a pilot study with a small sample size so that all the results must be viewed as indicative and should be replicated with a much larger sample.

**Table 1:** Principal components analysis for CS&S measures and ATE tasks after varimax rotation and retention of factor loadings ≥0.35.

CS&S measures and ATE tasks	Factors and variance explained (%)		
	1	2	3
	Literacy (22%)	Phonology (19%)	Visual (12%)
Writing words	**0.818**	0.379	
Rapid naming (Figure)	**–0.791**		
Reading accuracy	**0.787**	0.419	
Rapid naming (Number)	**–0.768**		
Writing pseudowords	**0.765**	0.370	
Reading fluency	**0.752**	0.408	
Alphabet task	**0.621**		
Word sequence	0.454		
ATE III-rhyme	0.416	**0.687**	
Non-word reading	0.413	**0.677**	
Auditory word discrimination		**0.665**	
Alliteration		**0.645**	
ATE I-alliteration		**0.634**	0.459
Rhyme detection	0.412	**0.629**	
ATE IV-rhyme		**0.604**	
Verbal number sequence backward		0.452	
ATE II-alliteration		0.358	
Syllable segmentation			
Shapes copying			
Figure rotation hit			**0.903**
Figure rotation error			**–0.761**
Figure order			**0.639**
Non-word repetition			0.490

Composite scores based on summed raw scores of writing (writing words + pseudowords), reading (reading fluency + reading accuracy + reading non-words), phonological (alliteration + rhyme detection + syllable segmentation), VWM (word sequence, non-words repetition, verbal number sequence backward), RN (RN of figures and digits) and, finally, ATE tasks (ATE I + ATE II + ATE III + ATE IV) were created (**Table 2**) in order to perform *t*-tests aimed at examining differences between academic low-achievers and non-low achievers in cognitive-linguistic abilities from the CS&S protocol and ATE tasks as well (**Table 3**).

**Table 2:** Descriptive characteristics of performance on CS&S measures and on ATE tasks on the basis of composite scores (*n* = 45).

Measure	Mean ± SD	Percentiles				
		*P*_25_	*P*_20_	*P*_15_	*P*_10_	*P*_5_
Reading	105.09 ± 28.689	94.00	90.20	81.80	63.80	27.20
Writing	30.09 ± 8.339	27.00	24.40	21.90	18.40	8.90
Literacy	135.18 ± 36.187	120.50	112.00	106.70	88.60	36.10
Phonological awareness	36.24 ± 4.488	33.50	33.00	32.00	30.00	25.00
Auditory word discrimination	18.62 ± 0.984	18.50	18.00	18.00	18.00	17.30
Verbal working memory	29.00 ± 3.090	26.50	26.00	26.00	25.00	24.30
Shapes copying	4.84 ± 2.132	3.00	2.20	2.00	1.60	1.00
Figure order	5.62 ± 1.134	5.00	5.00	4.00	4.00	4.00
Figure rotation error^a,b^	2.40 ± 3.129	3.00	4.80	6.10	7.00	8.70
Figures rotation hit	14.38 ± 6.065	9.00	8.20	7.00	6.00	3.60
Naming speed^b^	83.98 ± 18.165	90.50	94.80	99.10	105.80	126.30
ATE	32.16 ± 4.931	29.00	27.00	26.90	25.50	22.00

*^a^Original non-composite scores; ^b^Values represent percentiles 75, 80, 85, 90, and 95, respectively.*

**Table 3:** Descriptive characteristics (mean ± standard deviation) of performance on composite scores of CS&S measures (except auditory word discrimination and visual tasks) and ATE tasks in children identified as academic low-achievers compared to those considered typical learners.

	Mean ± SD			
Task	Non-low academic achievers (*n* = 32)	Academic low-achievers (*n* = 13)	*t*-test	*p*-value
Alphabet	25.09 ± 1.907	22.92 ± 3.774	1.974	0.680
Reading	119.00 ± 13.036	70.85 ± 28.151	5.915	0.000***
Writing	34.13 ± 3.260	20.15 ± 8.783	5.581	0.000***
Literacy	153.13 ± 14.788	91.00 ± 35.541	6.092	0.000***
Phonological awareness	38.22 ± 2.791	31.38 ± 4.214	6.391	0.000***
Verbal working memory	29.94 ± 2.850	26.69 ± 2.428	3.602	0.001**
Auditory word discrimination	18.88 ± 0.336	18.00 ± 1.633	1.916	0.079
Shapes copying	5.19 ± 1.839	4.00 ± 2.614	1.494	0.153
Figure order	5.84 ± 1.139	5.08 ± 0.954	2.138	0.038*
Figure rotation error	2.13 ± 3.077	3.08 ± 3.278	–0.923	0.361
Figure rotation hit	15.63 ± 5.615	11.31 ± 6.250	2.263	0.029*
Rapid naming	78.44 ± 11.733	97.62 ± 23.894	–2.762	0.015*
ATE	33.88 ± 3.966	27.92 ± 4.609	4.355	0.000***

Asterisks indicate significance (*p < 0.05, **p < 0.01, ***p < 0.001).

Analysis of individual data across the different domains was also conducted in order to derive individual profiles of performance and, thus, to determine in which domains a given at-risk student did and not did show abnormal performance. It was adopted the 25th percentile as a first criterion for deviance in the screening processes (**Table 4**). Poor literacy at the end of fifth grade was designated by performances 1 SD ≤ *M* on Literacy, a composite score including all reading and writing measures of the CS&S protocol (**Table 5**). Finally, logistic regression analyses were performed to explore the accuracy (sensitivity, specificity, and accuracy) with which collective measures, namely, the writing tasks of the CS&S protocol and the ATE tasks, as well as their combinations, predicted reading at the end of fifth grade (**Table 6**). All statistical analysis were conducted by using the SPSS software package, version 20.0 (SPSS Inc., Chicago, IL, USA) for Windows.

**Table 4:** Academic low-achievers composite scores at the beginning of the second grade on reading, writing, phonological awareness (PAs), verbal working memory (VWM), rapid naming (RN), and the alternative tool for educator’s (ATEs), and single scores of alphabet, auditory word discrimination (AWD), shapes copying (SHAPE), figure ordering (FIGOR), figure rotation error (ROTE) and figure rotation hit (ROTH) subtests (^**a**^scores ≤*P*_**25**_ or > *P*_**75**_ for ROT and RN; ^**b**^scores ≤*P*_**10**_ or > *P*_**90**_ for ROT and RAN; ^**c**^scores ≤*P*_**5**_ or >*P*_**95**_ for ROTE and NS).

	Low-achievers at the beginning of the second grade (A = ADHD symptoms)												
	04	05	06	10	18	19	22	24	27	30	32	41	43
Task (*P*_25_)			A		A	A				A	A		
Alphabet (24)	22^a^	26	19^b^	26	21^b^	21^b^	24^a^	26	25	24^a^	13	26	25
Reading (94)	95	80^a^	37^b^	53^b^	95	90^a^	71^a^	100	23^c^	82^a^	20^c^	91^a^	84^a^
Writing (27)	16^b^	27^a^	11^b^	20^a^	21^a^	27^a^	28	24^a^	8^c^	22^a^	2^c^	33	23^a^
Literacy (121)	111^a^	107^a^	48^b^	73^b^	116^a^	117^a^	99^a^	124	31^c^	104^a^	22^c^	124	107^a^
PA (34)	32^a^	30^a^	36	25^c^	33^a^	25^c^	37	34^a^	25^c^	30^a^	33^a^	36	32^a^
AWD (18.50)	18^a^	19	19	13^c^	19	19	19	18^a^	19	18^a^	18^a^	18^a^	17^c^
VWM (27)	31	28	27^a^	26^a^	28	25^b^	26^a^	28	27^a^	22^c^	24^c^	30	25^b^
SHAPE (3)	1^c^	7	7	7	4	2^a^	3^a^	7	3^a^	2^a^	1^c^	7	1^c^
FIGOR (5)	7	6	5^a^	4^b^	5^a^	4^b^	5^a^	6	5^a^	4^b^	5^a^	6	4^b^
ROTE (3)	1	1	7^b^	4^a^	9^c^	7^b^	0	0	0	7^b^	2	1	1
ROTH (9)	22	17	7^a^	6^b^	5^b^	3^c^	14	18	14	3^c^	12	17	9^a^
RN (90.50)	80	100^a^	132^c^	88	88	94^a^	79	91^a^	152^c^	110^b^	113^b^	71	71
ATE (29)	31	27^a^	35	22^c^	34	26^a^	27^a^	29^a^	19^c^	27^a^	30	32	24^b^

**Table 5:** Academic low-achievers’ composite scores at the end of the fifth grade on reading, writing, and literacy (^**a**^poor literacy status, i.e., scores 1 SD ≤*M*; ^**b**^scores ≤*P*_**25**_; ^**c**^scores ≤*P*_**5**_) and the true positives (TPs), true negatives (TNs), false positives (FPs), and false negatives (FNs) produced by CS&S writing tasks (W) and ATE tasks, separately and combined (W/ATE), administered at the beginning of the second grade as the second stage screening.

	Low-achievers at the end of the fifth grade (A = ADHD symptoms)												
	04	05	06	10	18	19	22	24	27	30	32	41	43
Task (*P*_25_)			A		A	A				A	A		
Reading (129)	134	119^b^	103^c^	121^a^	134	122^a^	124^a^	143	99^c^	134	113^b^	121^a^	161
Writing (32)	33	31^a^	31^a^	28^b^	24^c^	31^a^	29^b^	24^c^	26^c^	22^c^	28^c^	36	34^c^
Literacy (160)	167	150^a^	134^a^	149^a^	158^b^	153^a^	153^a^	177	125^a^	166	141^a^	157^b^	161
W	FP	TP	TP	TP	FP	TP	FN	FP	TP	FP	TP	TN	FP
ATE	TN	TP	FN	TP	TN	TP	TP	FP	TP	FP	FN	TN	FP
W/ATE	FP	TP	TP	TP	FP	TP	TP	FP	TP	FP	TP	TN	FP

**Table 6:** Prediction of PL status (Sens = sensitivity; Spec = specificity; Accur = accuracy) at the end of fifth grade according to logistic regression models 1, 2, and 3 based on one-stage screening with collective tools.

Models (1–4)	*B*	*SE*	Wald	*p*	TN	FN	TP	FP	Sens	Spec	Accur
**Model 1**
Writing	–0.225	0.094	5.746	0.017	34	0	7	4	100	89.5	91.1
ATE	–0.297	0.167	3.178	0.075							
Constant	12.957	6.040	4.602	0.32							
**Model 2**
Writing	–0.227	0.079	8.292	0.004	32	0	7	6	100	84.2	86.7
Constant	4.373	2.078	4.427	0.035							
**Model 3**
ATE	–0.287	0.106	7,381	0.007	26	1	6	12	85.7	68.4	71.1
Constant	6,947	3,057	5,165	0.023							

## RESULTS

The results of the first-stage universal screening based on CBMs identified 13 at-risk students. In the second stage of our two-stage screening process, some CS&S and ATE measures showed “ceiling effect” [more than 15% of respondents achieving the highest possible score scores (McHorney and Tarlov, 1995)], and a few significant outliers were found in the data. However, the method of PCA is relatively robust to these effects because assumes measurement without error, does not provide standard error and does not require distributional assumptions such as normality and homoscedasticity (Quinn and Keough, 2002; Jolliffe, 2005; Schmitt, 2011). Ceiling effects were observed in 10 variables: auditory word discrimination (AWD; 75%), syllable segmentation (69%), reading non-words (64%), ATE III (55%), alphabet (51%), alliteration and non-words repetition (29%), reading accuracy (20%), ATE I and ATE IV (18%). As for the PCA, the further analyses based on composite scores, which do not show ceiling effects, should not be affected by these effects.

Taken together, the presence of many coefficients ≥0.30 revealed by a correlation matrix containing all measures under investigation, jointly with the value of 0.675 of the Kaiser-Meyer-Olkin measure of sample adequacy (KMO value) and the statistically significant result of the Bartlett test of sphericity (*p* = 0.000), these results indicate that the data are suitable for factor analysis. A PCA suppressing coefficients smaller than 0.35, conducted for all CS&S variables and all four ATE tasks (totalizing 23 measures), revealed the presence of six components with an eigenvalue exceeding 1.0 which accounted for 75.84% of the data variability. However, Cattell’s scree test, used as a factor retention test, recommended extracting only three components which accounted for 53.1% of the variability. By looking at the commonalities among variables that more strongly correlate with one of the three factors it allows one to know what cognitive dimension each factor represents and, hence, to choose an appropriate name for each factor. Varimax is a method of rotating the component matrix which emphasizes differences in correlations (loadings) of the variables with each factor (maximizing high correlations and minimizing low ones) and was performed on these three components to give further support to the assumption that factors are indeed relatively independent from each other (Dancey and Reidy, 2007, pp. 469–472). Varimax rotation revealed the presence of a simple and clear structure of three components (**Table 1**), each showing a number of variables with specific and salient loadings. The few variables that loaded on two factors were allocated to the factor with which it had the highest loading.

Loadings on each of the three factors are outlined in **Table 1**. The first component, which can be assumed to represent the literacy dimension, accounted for 22% of data variance and had its highest loadings (between 0.70 and 0.80) on writing words (0.818), RN – figure (–0.791), reading accuracy (0.787), RN – number (–0.768), writing pseudowords (0.765), reading fluency (0.752), whereas its lowest loadings are found for alphabet task (0.621), and word sequence (0.454). For the second component, which represented the phonology dimension and accounted for 19% of data variance, the highest loadings (between 0.60 and 0.70) are found for ATE III (0.687), non-word reading (0.677), AWD (0.665), alliteration (0.645), ATE I (0.634), rhyme detection (0.629), ATE IV (0.604), and the two lowest loadings are for verbal number sequence backward (0.452) and ATE II (0.358). Finally, the third component, which represented the visual dimension and accounted for 12% of data variance, had its highest loadings for figure rotation hit (0.903), figure rotation error (–0.761) and figure order (0.639), and the lowest loading is for non-word repetition (0.490).

According to these results we could reduce the number of variables by using composite scores obtained from the sum of raw scores on conceptually related cognitive measures with the aim to facilitate analysis and interpretation of data, as well as to provide educators with a simple way to interpret children’s performance in a school context. Then, we grouped children’s responses in the composite scores (see Statistical Analysis in the Materials and Methods) of Reading, Writing, PA, VWM and, finally, ATE tasks that, jointly with AWD and visual measures presented in original single scores, are outlined in **Table 2** which contains mean scores, standard deviation and percentiles.

After performing ranking transformation, Spearman correlation analysis (CA) was conducted in all measures under investigation and showed that ATE tasks correlated with several composite scores from CS&S measures related to literacy and phonological abilities. From the highest to the lowest, correlations of ATE tasks with CS&S measures are: literacy (*r* = 0.595, *p* = 0.000), reading (*r* = 0.589, *p* = 0.000), VWM (*r* = 0.572, *p* = 0.000), PA (*r* = 0.548, *p* = 0.000), writing (*r* = 0.505, *p* = 0.000), RN (*r* = 0.447, *p* = 0.002), figures order (*r* = 0.423, *p* = 0.004), figure rotation hit (*r* = 0.368, *p* = 0.013). These correlations give further support to our hypothesis that ATE tasks are suitable for measuring phonological abilities collectively in classrooms.

**Table 3** provides the mean scores and standard deviation related to the measures described in the **Table 2** for those children identified academic low-achievers (*n* = 13) by the first stage of our two-stage screening procedure as well as for the remaining children not considered academic low-achievers (academic non-low achievers, *n* = 32). Since our sample size (*n* = 45) fits well with the central limit theorem, according to which sample sizes equal or greater than 30 are sufficiently large for concerns about unequal variance and non-normality (Sokal and Rohlf, 1987, p. 107), we conducted a *t*-test to compare performances on CS&S measures and ATE tasks of children designated as academic low-achievers by CBA measures and those children considered normal readers (**Table 3**). We used Levene’s test for inequality to verify whether variances between groups are equal and to decide what *t*-value to choose and, then, its significance. No significant difference in age was found between groups. As is outlined in **Table 3**, academic non-low achievers demonstrated significantly better performance than academic low-achievers on a number of C&S measures and in ATE tasks as well. Note that the greater *t*-values are observed for PA (*t* = 6.391, *p* < 0.001), Literacy abilities (*t* = 6.092), Reading (*t* = 5.915, *p* < 0.001), Writing (*t* = 5.581, *p* < 0.001) and ATE tasks (*t* = 4.355, *p* < 0.001), in this order, which were substantially greater than *t*-values for VWM (*t* = 3.602, *p* < 0.01), Naming Speed (*t* = –2.762, *p* < 0.05), Figure Rotation Hit (*t* = 2.263, *p* < 0.05), Figure Order (*t* = 2.138, *p* < 0.05).

An analysis of individual performance across the composite and individual measures of the CS&S protocol and the composite scores of ATE tasks, as described in **Tables 2 and 3**, was also conducted on the academic low-achievers (**Table 4**). This allows both the identification of at-risk students in the second stage and to derive individual profiles of performance (i.e., to determine in which domains a given at-risk student did and not did show abnormal performance) for which it was adopted norm-referenced cutoff points to determine deviance. As shown in **Table 4** the 25th percentile was the main criterion for deviance, but scores at or below 20th, 15th, 10th, and 5th percentiles were also included in the analysis. **Table 4** shows that CBAs, collectively administered by teachers in the classrooms as part of the first stage, was efficient in identifying children at risk for dyslexia. CBA measures have identified 13 children as struggling learners, from which 10 were found to perform below 25th percentile on reading tasks and 11 on writing tasks from the CS&S protocol. Performances on the Literacy composite score were found to be handicapped in 11 of the 13 academic low-achievers. From the non-literacy based tasks of the CS&S protocol, PA (10 of 13) and FIGOR (9 of 13) were the most frequently impaired in the academic low-achievers, followed by VWM (8 of 13), AWD, SHAPE and NS (7 of 13), ROTH (6 of 13), and ROTE (5 of 13). ATE tasks were handicapped in 8 of the 13 academic low-achievers thus comparably to VWM, the third most frequently impaired CS&S non-literacy based task. Children with ADHD symptoms, identified by the pedagogical team endorsements on the DSM-IV ADHD symptom checklist, is also shown in **Table 4**. Five of the 11 academic low-achievers also were identified as at risk for ADHD. It is worthy of noting that four of the five children with clear visuospatial difficulties (performing at or below 25th percentile in all three visual measures) were identified as at risk for ADHD.

From now on we will focus our analyzes on the accuracy with which our collective screening tools, namely, CS&S writing tasks and ATE tasks, predict poor literacy status at the end of fifth grade (literacy scores 1 SD ≤*M*) as shown in **Tables 5 and 6**. Since participant/variable ratio recommended in books for regression analysis range from 15 to 40 participants per variable (Dancey and Reidy, 2007), we considered that our sample size fits well within these requirements once we have only two variables under focus, i.e., composite scores of the collectively administered writing and ATE tasks. We conducted regression analysis with Writing and ATE composite scores, both separately and together, as predictors. Together Writing and ATE tasks accounted for nearly half the variation of literacy abilities at the end of fifth grade (adjusted *R*^2^ = 0.486), *F*(2,38) = 19.886, *p* < 0.001. Writing has a larger absolute standardized coefficient (0.651) compared to ATE tasks (0.118), thus reflecting its higher contribution to the model. Alone, Writing accounted for 48.8% of the variance [*F*(1,39) = 39.107, *p* < 0.001] in literacy abilities at the end of fifth grade, against 16.3% of the variance explained by ATE tasks [*F*(1,39) = 8.777, *p* < 0.01].

We performed logistic regression to predict normal reader and poor literacy status at the end of fifth grade (PL, i.e., children with performances ≤1 SD below the mean on literacy tasks). To maximize TPs and limit FPs we explored the utility of several classification cutoffs in addition to the standard 0.5 classification cutoff of the SPSS according to the suggestion of Neter et al. (1996). By adjusting the cutoff value at either 0.2 or 0.1, the accuracy of the three logistic regression models based on one-stage screening in the entire group (*n* = 45) using Writing and ATE collective screening tools, either separately or combined, as predictors of PR at the end of fifth grade, are described in the **Table 6**. Model 1, combining both CS&S writing and ATE tasks, and Model 2, based only on writing tasks, both resulted in a sensitivity of 100% and specificities of 89.5 and 84.2%, respectively. Model 3, based only on the ATE tasks resulted in a sensitivity of 85.7% and specificity of 68.4%. The Hosmer–Lemeshow test showed small and non-significant Chi-squared values indicating goodness of fit for the logistic regression models.

## DISCUSSION

The most common way to implement RTI in the schools is the reliance on a single-stage universal screen to determine children at risk for learning disabilities and to provide them with Tier 2 interventions. Despite the advantages of being efficient, requiring only a brief, one-time assessment which provides objective information about the academic status of students, a single-stage universal screening often overidentify at-risk status (Fuchs et al., 2012; Gilbert et al., 2012). There is evidence that use of a second stage screening reduces the number of FPs and, hence, the number of students in need of Tier 2 intervention by two thirds, reducing the total cost of approximately $260,000, considering a universe of 200 at-risk children, to approximately $96,500 (Fuchs et al., 2012). However, in a two-stage screening process both first and second stages are based on individual assessments what implies in additional time, personnel and financial resources, even more if we take into account the expense of tutoring interventions in Tiers 2 and 3. Taken together, even in a RTI implemented on the basis of a two-stage screening process these aspects make the implementation of RTI in Brazil a challenging endeavor if we consider the public funding limitations.

One of the ways to reduce the costs of both one-stage and two-stage screening procedures is the use of screening tools that are able to be administered collectively (Andrade et al., 2013). The main purpose of this pilot study was to evaluate the accuracy of two collective screening tools in identifying children at risk for dyslexia in the beginning of the first grade and to predict poor reading status at the end of fifth grade. Writing tasks of the collective version of the CS&S protocol and a non-literacy based tool for the collective assessment of the phonological abilities based on phonological judgments by matching figures and figures to spoken words (ATE tasks), were tested as collective screening tools in the context of both a one-stage and a two-stage screening processes.

By conducting a PCA analysis with varimax rotation on the CS&S measures we found the presence of a simple and clear structure of three components, namely, a literacy dimension represented by measures of reading and writing abilities, a phonological dimension mainly represented by tasks involving phonological processing and, finally, a third dimension representing visuospatial processing. Overall, the results above indicate that the tasks proposed by CS&S protocol are, indeed, representing the cognitive-linguistic or conceptual dimensions which they are intended to measure.

More relevant to our investigation is the finding that three of the collectively administered ATE tasks presented high loadings on the phonology factor (or phonological dimension) and correlated with several composite scores from CS&S measures related to literacy and phonological abilities. More specifically, ATE III showed the highest of all loadings on phonology factor, followed by ATE I being the fifth highest loading and by ATE IV being the seventh highest loading. Secondly, an analysis of individual performance across the composite and individual measures of the CS&S protocol and the composite scores of ATE tasks, allowed both the identification of at-risk students and to derive their individual profiles of performance. From the 13 children identified as struggling learners by CBA measures administered collectively by teacher in the classrooms, 11 were found to be handicapped in literacy abilities and 10 were handicapped in phonological abilities, from which eight were also handicapped in ATE tasks, this last the third most frequently impaired from all non-literacy based task.

Moreover, individual profiles of linguistic-cognitive deficits in academic low-achievers are consistent with literature in that although a phonological processing deficit is the main endophenotype associated with literacy acquisition difficulties it may not be sufficient to fully explain dyslexia (Peterson and Pennington, 2012). Firstly, we found that attention deficits are present in five of the 11 children identified as at risk by the second-stage screening (**Table 4**) who were later part of the seven poor literates at the end of fifth grade (**Table 5**). Additionally, two of these poor literates had normal phonological processing, while both were severely impaired in naming speed and one of them was identified as at risk for ADHD. Therefore, their literacy difficulties could be explained only on the basis of naming deficits, consistently with the double-deficit hypothesis of dyslexia (Wolf and Bowers, 1999; Boada et al., 2012). Individual analysis also revealed that all children identified as at risk for ADHD were impaired on RN and four were clearly impaired in VWM and in all three visuospatial measures (figure order, figure rotation error, and figure rotation hit). These findings are strikingly consistent with the notion that inattention symptoms are associated with weaknesses in naming speed and working memory (Shanahan et al., 2006; Lui and Tannock, 2007; Marzocchi et al., 2008; Arnett et al., 2012), and also consistent with evidence that tasks involving visuospatial manipulations are specially sensitive to inattentive behavior (Martinussen et al., 2005; Willcutt et al., 2005; Castellanos et al., 2006).

Taken together, these results support our proposal that the collective administration of the ATE tasks can substitute the individually administered phonological processing tasks from the CS&S protocol. A third important finding is regarding the collectively administered writing tasks of the CS&S protocol, namely, words and pseudowords, which represented the highest and the fifth highest loadings on the literacy factor, respectively, thus suggesting that this task also has the potential to be used as a literacy-based screening tool. In fact, Snowling and Hulme (2012) argue that although few causal theories have addressed the complex relations between reading and writing development it is clear that most cognitive mechanisms underlying these abilities are shared, so that reading difficulties will be associated with writing difficulties most of the time. These authors further argue that once spelling demands explicit knowledge of the orthographic structure of words its development depends in large part on reading experience which, in short, “gives information in how phonology maps to orthography”; in this sense, spelling is protracted in relation to reading which normally begins to significantly influence spelling around the second year of reading instruction (Snowling and Hulme, 2012, p. 596). This rationale is consistent with the fact that spelling and writing problems are often more severe and more persistent than reading problems in people with dyslexia and our findings fit well within this framework.

Logistic regression showed that both collective screening tools under investigation, namely CS&S writing subtests and ATE tasks, used as one-stage universal screening tools (in isolation or combination) were statistically significant predictors of literacy abilities at the end of fifth grade. In combination, Writing and ATE tasks (Model 1) accurately classified 91.1% of poor readers at the end of fifth grade, yielding sensitivity of 100% and specificity of 89.5%. Writing alone (Model 2) classified 86.7% of poor readers with 100% of sensitivity and 84.2% of specificity, whereas ATE tasks accuracy (Model 3) was 71.1% with sensitivity of 85.7% and specificity of 68.4%.

Of relevance is the finding that a first-stage universal screening based on CBA measures collectively administered by teachers in classrooms showed to be of great utility identifying 13 academic low-achievers, corresponding to 29% of the whole sample. From the 13 children identified as academic low-achievers by CBA measures 11 were identified as at risk for dyslexia in the second-stage universal screening. Moreover, all seven students identified as poor readers at the end of the fifth grade were academic low-achievers screened by CBA measures in the beginning of the second grade. Although teachers’ judgments of student literacy abilities are too subjective and show significant variability (Madelaine and Wheldall, 2005) these results are consistent with the notion that well clearly defined pedagogical assessments administered by teachers in classrooms have the potential to be used as a first-stage universal screening. CBA measures has reduced the number of children referred to Tier 2 interventions by 15.4%, and could be used as first-stage universal screening followed by ATE tasks and Writing tasks as second-stage screening in a two-stage screening procedure.

There are several study limitations and admonitions regarding the interpretations of our study. The first and most important is the small sample size which imposes serious limitations regarding the generalizability of our results. Therefore, our results should be interpreted in the context of a pilot study which took care of controlling homogeneity with respect to age, socioeconomic status and pedagogical approaches, so that our results should be consistent with studies on larger samples in order to be considered as a valid pilot study worthy of being conducted in larger, representative samples. Secondly, once we do not have detailed information regarding the general non-verbal cognitive abilities of these children it remains unclear whether differences between groups are due to differences in general cognitive abilities, e.g., IQ or even overall processing speed. However, only students with no exclusionary factors participated in the study. We also have examined separately those tasks traditionally associated with executive cognitive functions and noted that they were not significantly correlated with reading abilities neither showed high loadings on literacy factor. These tasks are Verbal Number Sequence Backward, which measures the ability of mental double-tracking of the memory and the reversing operations (Gathercole et al., 2004; Lezak et al., 2004), Figure Order, Rotation Error (and Figure Rotation Hit) which measure visual short-term memory and visuospatial abilities, respectively, thus tapping visual memory functions different from those assessed by verbal-auditory measures (Snow, 1998; Rosselli et al., 2001; Kyttälä, 2008) and tapping attention as well (Marzocchi et al., 2008). Therefore contributions of these abilities to the differences between groups and the relationships between variables should be minimal. The third limitation is regarding to the measures used to define poor reading status at the end of fifth grade, which did not include a subtest of reading comprehension. To minimize this limitation we also took into account the fifth grade CBA measures administered by teachers and the interviews with the pedagogical team to confirm the academic status of those children identified as poor readers. The fourth limitation is with respect to the cutoff point to designate low-achievement (1 SD ≤*M*) which could be considered too lenient. However, this cut point was used in relation to the entire sample, which also included academic low-achievers, so that it defined a more stringent cutoff that if it were used in relation to only those students with normal literacy abilities.

Overall our results provide empirical support for the notion that further studies on developing and testing of collective screening tools for the identification of children at risk for learning disabilities should be encouraged.

## Conflict of Interest Statement

The authors declare that the research was conducted in the absence of any commercial or financial relationships that could be construed as a potential conflict of interest.
